# Development and validation of a scale to assess attitudes of health care providers towards persons affected by leprosy in southern India

**DOI:** 10.1371/journal.pntd.0006808

**Published:** 2018-09-25

**Authors:** Govindarajulu Srinivas, Shuba Kumar, Rani Mohanraj, Geethalakshmi Sekkizhar, Thirumugam Muthuvel, Vivek Lal, Burkard Koemm, Christa Kasang

**Affiliations:** 1 Department of Epidemiology, The Tamil Nadu Dr. M.G.R. Medical University, Chennai, India; 2 Samarth, Chennai, India; 3 Samarth, Chennai, India; 4 The Tamil Nadu Dr M.G.R. Medical University, Chennai, India; 5 Epidemiology Unit, Global Data Research Center, Hyderabad, India; 6 German Leprosy and TB Relief Association, Kolkata, India; 7 Deutsche Lepra- und Tuberkulosehilfe e.V., Wuerzburg (DAHW), Germany; 8 DAHW, Wuerzburg, Germany; University of Tennessee, UNITED STATES

## Abstract

**Introduction:**

Assessment of attitudes of health care professionals is important as negative attitude could constitute a major deterrent to care-seeking by persons affected by neglected tropical diseases (NTDs) such as leprosy. Leprosy continues to pose a major disease burden in India with an annual new case detection rate of 10.17 per 100,000 population. This paper reports on the development and validation of a culturally appropriate scale to measure attitude of health care providers (HCPs) towards persons affected by leprosy in Tamil Nadu, India.

**Methodology/Principal findings:**

The Affective, Behavioural and Cognitive (ABC) model of attitudes guided the development of the scale. Steps in scale development included qualitative interviews and focus group discussions with medical officers and paramedical staff selected from high prevalence districts in Tamil Nadu, India which informed the development of the draft scale. Reviews of existing attitude questionnaires in related areas further contributed to scale development and together helped to generate a large pool of items which was then subjected to Thurston’s scaling method for selection of items from this pool. Face and content validity were obtained, following which internal consistency and test, re-test reliability were assessed. Scaling exercise resulted in 11 items being discarded from an initial pool of 38, owing to the poor agreement among experts regarding relevance. Face and content validity were good with experts endorsing relevance and applicability of items. The intra-class correlation coefficient (ICC) for test re-test reliability of the 27 item scale was 0.6 (95% CI: 0.20–0.78) indicating marginal intra-class correlation. The overall Cronbach’s alpha was 0.85 while the alphas for each of the affective and behavioural components was good at 0.78 and 0.69 respectively indicating a good degree of consistency and homogeneity between items but the alpha for the cognitive component was low at 0.53.

**Conclusions:**

The ABC model of attitudes guided the development of the scale, ensured a mix of 27 items tapping into the three domains of Affect, Behaviour and Cognition which best explained the attitude construct. With good validity and alphas for each of the affective, behavioural components and overall alpha estimates, this scale can be a valuable tool to provide accurate estimates of the true attitudes held by HCPs. This, in turn, would be useful to obtain insights for appropriate intervention programmes that would help change negative attitudes of HCPs towards persons affected by leprosy. With some adaptations, the scales can be validated for other NTDs as well.

## Author summary

Leprosy is an infectious disease caused by the *Mycobacterium leprae* and is one of the major causes of preventable disability. Early diagnosis and prompt treatment of all new cases of leprosy remain the key strategies for leprosy control as it would prevent nerve damage, disability and reduce the transmission of the disease. People affected by leprosy often experience severe stigmatization because of an adverse social judgment about the disease or its disabling consequences. This neglected tropical disease continues to pose a major disease burden in India. Despite the availability of health facilities there continue to be barriers towards leprosy diagnosis and early treatment. Assessment of attitudes of health care professionals is important as negative attitude could constitute a major deterrent to care-seeking by persons affected by leprosy. Researchers developed and validated a culturally appropriate scale to measure attitudes of health care providers towards persons affected by leprosy in Tamil Nadu, India. The scale would be useful to obtain insights of attitudes of health care professionals to plan appropriate programmes that would help to promote positive attitudes of healthcare providers towards persons affected by leprosy.

## Introduction

Leprosy continues to pose a major disease burden in India with an annual new case detection rate of 10.17 per 100,000 populations. An average of 0.13 million new leprosy cases are detected every year in the country [1]. Despite the availability of health facilities there continue to be barriers towards leprosy diagnosis and early treatment. People affected by leprosy often experience severe stigmatization as a result of an adverse social judgment about the disease or its disabling consequences [2]. Their family members in turn experience negative attitudes, face social isolation and other discriminatory practices [3–5]. As a consequence, patients and their families often feel forced into stopping treatment for fear of being exposed, thereby contributing to increased morbidity and risk of disability [6]. The problem can be further compounded by negative attitudes of Health Care Providers (HCPs) which could constitute a major deterrent to care-seeking by patients. In a study carried out in Mumbai, it was found that 75% of general physicians who were untrained in the management of leprosy believed that isolation and treatment of leprosy patients was necessary, while 59% were opposed to social integration of leprosy patients even after complete cure [7].

Attitudes and beliefs about leprosy, are shaped by factors like knowledge about the disease, opportunities to interact with persons having the condition, influence of cultural stereotypes about leprosy, influence of media, familiarity with institutional practices, religion and past restrictions [8]. Positive attitudes held by HCPs towards persons affected by leprosy facilitate better access and utilization of health services [9]. Furthermore, if HCPs are seen as being willing and are comfortable treating persons affected by leprosy, it could lead to improved quality of care and enhance the value of these services [10]. By their training and knowledge about the disease, HCPs are usually expected to bear positive attitudes towards persons affected by leprosy [9]. Unfortunately, this may not always be the case. Croft and Croft report that the negative attitudes of health care workers acted as a block to the delivery of holistic health care for persons affected by leprosy and also contributed to reinforcing harmful traditional beliefs about leprosy in the community [11]. Reports also suggest that medical doctors too have been known to avoid providing care to persons affected by leprosy to the extent of even refusing to treat them [12]. The resultant sense of discrimination and shame that persons affected by leprosy face, invariably, causes them to avoid seeking care.

Persons affected by neglected tropical diseases (NTDs), such as leprosy, can experience stigma. In addition to the direct effects of societal stigma, persons suffering from NTDs face several forms of structural stigma, such as lack of resources allocated to this neglected group and low training levels and negative attitudes amongst health care staff. This, in turn, affects the availability, quality and uptake of treatments offered, resulting in poor treatment outcomes and persistent stigma. Negative attitudes amongst HCPs lead to low training levels, poor management resulting in poor prognosis & treatment outcomes, high visibility of illness [13,14].

Given that, persons affected by leprosy could also develop serious disabilities; that there are barriers towards diagnosis and treatment of leprosy attributable to stigma, the need to develop/modify and validate a culturally specific scale to measure the attitudes of HCPs towards persons affected by leprosy assumes significance. Such a validated scale will provide critical information on how HCPs perceive persons affected by leprosy thereby enabling appropriate interventions aimed at changing negative attitudes.

### Conceptual framework

The Affective, Behavioural and Cognitive (ABC) model of attitudes guided the development and selection of items for this scale [15]^.^ According to this model, attitude is comprised of 3 components- affective, behavioural and cognition. The affective component refers to emotional reactions individuals have towards the attitude object; the behavioural component refers to the way individuals behave when exposed to the attitude object and the cognitive component refers to an individual’s beliefs and knowledge about the attitude object.

A web search revealed 11 studies that assessed the knowledge, attitudes and practices of health care providers towards persons affected by leprosy. Most of these studies had been carried out in African countries [8,9,12,16–20] with three that had been undertaken in India [21–23]. Based on our review, we discovered that barring a few items, the majority of the items in the different scales were more representative of knowledge and practice rather than attitude thereby justifying the development of such a scale. The attitude items from these different scales used in the 11 studies were listed and served as the item pool from which- following information generated from the qualitative work- we made decisions on the ones to retain. The initial pool of items from these studies were examined for equivalence. Beaton et al. (2000) guidelines on cross cultural adaptation (to look for semantic, conceptual, experiential and idiomatic equivalence) of self-report measure guided us in this process. For example, one of the items that we did not include was “cause of leprosy is because of witches”. This was because the concept of ‘witches’ is not integral to the Indian culture [24].

## Materials and methods

### Ethics statement

Permission to conduct the study was obtained from the Directorate of Public Health & Preventive Medicine (DPH&PM) and the research protocol was approved by the Institutional Review Board of GLRA, India. Willing participants were required to sign an informed consent form. The study was carried out during the period April 2015 –March 2016.

A list of districts in the state of Tamil Nadu, which showed a high number of cases of leprosy were obtained from the DPH&PM,Chennai. The districts of Villupuram, Kancheepuram, Tiruvannamalai and Tiruvallur were some of the districts located relatively close to Chennai that was selected from this list. For the year 2013–2014, a total of 353 new cases of leprosy were reported in the district of Villupuram. Similarly, for the districts of Kancheepuram, Tiruvannamalai and Tiruvallur it was 160, 176 and 140 new cases respectively for the same time period. A list of primary health centers (PHCs) in each of these selected districts was also obtained from the DPH&PM and three PHCs were purposively chosen for the study. The Medical Officers (MOs), Health Inspectors (HIs) and Village Health Nurses (VHNs) in each of these PHCs were then contacted and their permission sought to participate in the study. Each of these cadres of HCPs, play important roles in the management of persons affected by leprosy. Thus, while both the HIs and VHNs assist in case detection in the community and in educating people about leprosy, etc., the MOs are involved in providing direct clinical services to the persons affected by leprosy. We also carried out interviews with persons affected by leprosy in order to understand their experiences about accessing care and any discriminatory behaviors they may have faced from HCPs.

For the qualitative component we included three PHCs from two districts namely Villupuram and Kancheepuram while for questionnaire validation we contacted as many HCPs from the above list who were willing to participate in the study. The scale development process was divided into two parts. In part 1, we carried out the qualitative Semi Structured Interviews (SSIs) and Focus Group Discussions (FGDs) for the purpose of item generation, followed by the scaling exercise and in part 2 we undertook the validation of the questionnaire.

### Part I

#### Qualitative interviews

A total of 10 SSIs were carried out with HIs and MOs (5 in each category) and two FGDs were conducted with the VHNs (10 in a group) selected purposively from PHCs in the districts of Kancheepuram and Villupuram with the aim of exploring their attitudes and beliefs about working with persons affected by leprosy. Separate SSI and FGD guides were developed, and new issues that emerged during the interviews and FGDs were explored. Examples of some of the questions asked were:

“What do you see as some challenges in working with leprosy patients and how do you deal with this?” (Probe: concerns regarding being at risk or becoming infected, issues concerning stigma)

“How much do you think issues concerning stigma come in the way of patients seeking care in general health care settings, please describe” (Probe: patient fears of being stigmatized/ discriminated against in the health facility, in the community)

“Do you believe that patients with leprosy face discrimination in health care settings, if so what form does this discrimination take and why do you think this happens?” (Probe: fears of contagion, whether they feel disgust at having to treat such patients etc.)

Only three persons affected by leprosy gave consent to participate in the SSIs. The guide meant for this group addressed issues such as their understanding of leprosy, how big a problem they think it is, at what stage do patients usually seek care from a doctor, their experiences of healthcare seeking, their perceptions on the attitudes of HCPs towards persons affected by leprosy and issues of stigma and discrimination they may have faced.

All the SSIs were carried out by SK and RM, both of whom are experienced researchers trained in qualitative and quantitative research methods. The FGDs were carried out by a senior research officer supported by either SK or RM. This qualitative component helped in the development of the scale, thereby strengthening its content validity. All interviews and FGDs were conducted in privacy in the respective PHCs to which the HCPs were attached and were audio-recorded after obtaining informed consent from the participants.

A framework analytical approach was used to analyze the qualitative data [25] with the specific focus on the affective, behavioral and cognitive components of the attitude construct. Thus, deductive coding was manually performed on each of the interviews and FGD transcripts independently by SK and RM with the aim of specifically looking for themes that captured the affect, behaviour and cognition components. Simultaneously, SK and RM also reviewed the list of attitude items we had initially selected following our review of the different attitude scales and examined these in the light of information generated through the qualitative interviews and FGDs.

#### Scaling

The initial draft scale was subjected to the process of Thurstone’s equal interval scaling to determine how well the items in the construct ‘hung’ together [26]. The scale was given to 11 experts, from the field of social sciences, community medicine and leprosy who were asked to rate each item on a scale of 1–11 where 1 = extremely unfavorable attitude towards persons affected by leprosy and 11 = extremely favorable attitude towards persons affected by leprosy. The ratings from the 11 individuals for each item were analyzed and median and inter-quartile ranges (IQR) for each item were computed to determine the level of agreement between them on each item. One statement from each of the 11 medians was selected. If there were more items with the same median value, the statement with the least inter-quartile range was selected as this statement had the least variability among the judges/experts, meaning that there was good agreement among the judges regarding the ratings given to the item. We took care to ensure that the final items that were selected included a good representation of the affective, behavioural and cognitive component of attitudes towards persons affected by leprosy.

### Part II

#### Validation of the questionnaire

Upon completion of Part I of the study, a one-day workshop consisting of the experts in the field of leprosy and persons affected by leprosy was held to review the scale which helped to assess its face and content validity. They checked if items reflected what they intended to measure. Experts in the field of leprosy assessed for content validity of the 27 items by reviewing whether the items were representative of the theoretical domains of affect, behaviour and cognitive components of attitude. This scale was designed as a self-rated questionnaire that HCPs could themselves rate. All items were framed as statements with a response format set on a 4-point continuum, namely, “Strongly Agree, Agree, Disagree and Strongly Disagree. The experts reviewed each item for its relevance and usage among providers in Indian health settings. Additionally, their comments on its comprehensiveness were also obtained. Based on their feedback and inputs, necessary revisions were made, and the scale was translated into Tamil, the language of communication in Tamil Nadu. This was done by members of the research team. The translation aimed to achieve semantic equivalence and ensure that the meaning of the original question was not lost during translation. The translated version was then back-translated by an independent person who was not part of the team, compared for equivalence with the English version and made ready for the reliability assessments. The reliability assessments were carried out in selected PHCs.

*Sampling*. The selection of PHCs was done purposively but was broadly based on the number of cases of leprosy being treated in that PHC. More important the HCPs had to be willing to participate in the reliability procedures. A minimum of one year of work experience in a PHC served as the eligibility criteria for selection of MOs, HIs and VHN. These HCPs were met with during their weekly review meetings and informed about the study. Those who gave informed consent were given the attitude scale which they had to complete and hand over to the study team. It was decided to administer the scale to a minimum of 50 HCPs based on feasibility.

*Reliability Assessments*: *Test-retest Reliability*, *Internal consistency (Cronbach’s alpha)*. The test re-test reliability of the scale was assessed by giving it to a sample of HCPs from PHCs located in two different districts, namely Kancheepuram and Tiruvallur. The HCPs included a mix of MOs, VHNs and HIs. After a gap of 10–12 days, the same HCPs were re-contacted and asked to rate it again. Test re-test reliability was assessed by calculating the intra-class correlation coefficient (ICC) for responses obtained at the two-time points, where 0.60 was considered marginal, 0.70 acceptable and anything over 0.80 was considered good correlation [27]

To assess how well the items measured different aspects of the same latent concept *(internal consistency)*, we assessed the correlation between items. A Cronbach’s alpha greater than 0.6–0.7 was indicative of good internal consistency [27].

## Results

### Key findings from qualitative interviews/FGDs with HCPs

Five MOs and five HIs participated in the qualitative interviews. While three MOs and three HIs were from Villupuram, two MOs and two HIs were from Kancheepuram. One MO and three HIs were women; all the rest were men. The MOs had all completed their MBBS degree. While four of the HIs held post-graduate degrees,one had only completed schooling (12 years). Two FGDs were carried out with the VHNs, one in Kancheepuram and one in Villupuram. The VHNs were all women who had completed their schooling and were trained in community health services.

As far as the MOs were concerned, they regarded leprosy like any other disease and were knowledgeable about its modes of transmission and management. They spoke of setting an example to others by being good role models so that other cadres of HCPs would learn from them and behave accordingly. The VHNs and HIs also had good knowledge about the disease but expressed the need for better understanding of its modes of spread and the side effects of treatment. They mostly spoke of the need to care for these patients and categorically denied any discriminatory behavior by any category of HCPs. Some said that such behavior had been present earlier but was no longer evident while others spoke of harboring some fears in the beginning which they got over once they started caring for patients. All the HCPs however, believed that stigma towards leprosy patients was very much present in the community. This was evident by the fact that patients did not want to be seen taking treatment and tried to hide their condition. They would specifically request the VHN and HIs to keep their disease a secret as they feared being isolated by the community.

The general opinion was that as the disease was identified early both because of better awareness of its symptoms by the community and because of proactive efforts made by VHNs and HIs, the disease rarely progressed to the stage of severe ulceration. Consequently, patients were diagnosed and treated early. Very few patients with severe ulceration and disabilities—which could give rise to both revulsion and fear—were seen by HCPs. A few VHNs opined that sometimes steps taken by them towards protecting others were misinterpreted and led to people affected by leprosy feeling offended and believing that they were being discriminated against.

Several issues emerging from the qualitative interviews were reflected in the items drawn from the scales used in the 11 studies. For example, one MO said that when he initially started working with leprosy patients he feared contracting the disease but over time he overcame the fear, “*Earlier I used to fear whether I too would develop leprosy*. *Now one year*, *I have been working in leprosy*. *Now I feel confident*. *In the beginning*, *I had this fear but later it was not there”* (PHC MO- Kancheepuram). The corresponding attitude item we selected was, ‘I am concerned with getting infection from patients with leprosy when I treat them’. Similarly, an HI indicated that seeing patients present with pus and ulcerative wounds was distressing, “*if they are having pus then we would feel like a kind of something*. *But what I can do about that*? *I would just tell them how to prevent from getting pus”* (PHC HI- Kancheepuram). The corresponding item we selected was, ‘There is a sense of revulsion when seeing a leprosy patient with ulcers’. The VHNs during the FGD said, *“As we treat any other patient*, *we also treat the leprosy patients in the same way*. *We don’t isolate leprosy patients and treat them separately”* (FGD VHN-Kancheepuram). One MO echoed the same issue when he said, “*I am seeing the leprosy patient as normal as how I see other patients*. *We practice precautionary measures*, *like wearing gloves* (PHC MO 3, Villupuram). The corresponding item we selected was. ‘It is possible to manage leprosy like any other disease in the general health service’. An HI described feeling a sense of satisfaction working and helping persons affected by leprosy, *“I feel good because when we identify cases and provide them treatment*, *it gives a good feeling until now*, *there were many people*, *who had no knowledge about this disease at all*, *and they had remained without taking any kind of treatment*. *So*, *when we identify cases and put them on treatment*, *it gives a satisfactory sort of feeling”* (HI Kancheepuram). The corresponding item we selected was, *‘I get a sense of satisfaction when I treat patients with leprosy’*. Thus the information derived from these qualitative interviews and FGDs served to corroborate the items we selected to constitute this attitude scale. We also took care to bring in both positive and negatively worded statements.

### Key findings from qualitative interviews with people affected by leprosy

All three persons affected by leprosy said that hospital staff treated them well, were supportive and had guided them to seek care early. They all said that the stigma they faced was mostly from the community and from family members as people feared contracting the disease. They therefore tended to isolate themselves to avoid being hurt and humiliated.

*“Yes*, *I take my medicines regularly*. *For my health problem*, *I should only take medicines regularly*. *Otherwise everybody will make fun of me*. *But some of them will say she has leprosy*. *So*, *we should not touch her… Yes*. *That’s why I am scared…*. *I did not say to anybody*. *My husband knows*, *and my brother knows*, *because he has also got this disease”* (person affected by leprosy.1 Kancheepuram).

*“In my house*, *nobody knows that I have this disease*. *They know that I am having medicines*. *I said I was not well and so am taking these tablets*. *I will get hurt so I did not want to reveal*. *I thought that let it be with me itself…*.. *But the doctor and the people in hospital they took care well*. . . . *I had the fear whether the doctor would scold me or discriminate me*. *But by god’s grace neither the doctor nor anybody else in the hospital treated me badly I had the fear that they might discriminate me but no one did”* (person affected by leprosy. 2 Kancheepuram).

#### Scaling

Scaling revealed a good range of items with varied median values ranging from 1–5 and then again from 8–11. There were, however, none with median values of 6 and 7. Only one item had a median value of 11. For all other median values there were a minimum of two to a maximum of seven items. In all such cases where there was more than one item with the same median value, those with the least IQR were selected, as this had the least variability among the judges/experts. Scaling exercise resulted in 11 items being discarded from an initial pool of 38, owing to the poor agreement among experts regarding relevance.

Tables 1, 2 and 3 present the set of items under each of the three components (Affective, Behavioural, and Cognitive) that were selected following the scaling exercise along with their median and IQR values. Thus, the Affective component had 10 items, the Behavioural component had 8 items and the Cognitive component had 9 items.

**Table 1:** Affective component items (10 items).

S. No	Selected Items	Median	IQR
2	Nursing of leprosy patients is a dirty job	2	2
4	People with leprosy should live apart from other people	2	1
5	There is a sense of revulsion when seeing a leprosy patient with ulcers	3	1
8	Leprosy patients with ulcers and deformities must be isolated	3	2
10	I am concerned with getting infection from patients with leprosy when I treat them	4	2
17	Dressing for a leprosy patient is not disgusting most of the times	8	2
18	Working in a leprosy hospital is one of the best ways of exhibiting humanitarian nature	8	2
19	If someone in my family has leprosy I would not mind talking about it to my friends	8	2
20	I get a sense of satisfaction when I treat patients with leprosy	9	1
21	I would be supportive of a person who has leprosy	9	1

1 = extremely unfavorable and 11 = extremely favorable

**Table 2:** Behavioural component items (8 items).

S. No	Selected Items	Median	IQR
7	At the hospital, I prefer to avoid touching someone with leprosy if possible	3	2
12	I prefer that dressing for leprosy wounds are carried out in a separate clinic	4	3
13	Staff and health care providers should be notified when a patient with leprosy comes for treatment	5	2
15	I would take special care (like wearing gloves and masks) when treating patients with leprosy	5	4
23	It would be better to treat leprosy patients in general hospitals instead of ‘special’ leprosy hospitals	10	1
24	I have no problem in treating leprosy patients with deformities or ulcers	10	1
25	I am willing to be involved in diagnosing/managing leprosy cases at my health care Facility	10	1
26	I don’t mind working in an all leprosy hospital	10	1

1 = extremely unfavorable and 11 = extremely favorable

**Table 3:** Cognitive component items (9 items).

S. No	Selected Items	Median	IQR
1	I think leprosy is a curse and a punishment	1	1
3	Children of leprosy patients invariably develop leprosy	2	2
6	Most workers in leprosy hospitals usually contract the disease	3	2
9	Deformities are an inescapable consequence of leprosy	3	2
11	The commonest presentation of leprosy is deformed limbs	4	2
14	There are no career prospects as far as nursing practice in leprosy hospital is concerned	5	3
16	It is possible for me to have leprosy if I treat leprosy patients	5	3
22	It possible to manage leprosy like any other disease in the general health service	9	3
27	Leprosy is curable	10	3

1 = extremely unfavorable and 11 = extremely favorable

Items selected following scaling:

Thus, following scaling, the total number of items in the scale was reduced to 27 *(Box 1).*

**Box 1: HCP attitudes towards persons affected by leprosy**

1. I think leprosy is a curse and a punishment
2. Nursing of leprosy patients is a dirty job
3. Children of leprosy patients invariably develop leprosy
4. People with leprosy should live apart from other people
5. There is a sense of revulsion when seeing a leprosy patient with ulcers
6. Most workers in leprosy hospitals usually contract the disease
7. At the hospital, I prefer to avoid touching someone with leprosy if possible
8. Leprosy patients with ulcers and deformities must be isolated
9. Deformities are an inescapable consequence of leprosy
10. I am concerned with getting infection from patients with leprosy when I treat them
11. The commonest presentation of leprosy is deformed limbs
12. I prefer dressing for leprosy wounds are carried out in a separate clinic
13. Staff and health care providers should be notified when a patient with leprosy comes for treatment
14. There are no career prospects as far as nursing practice in leprosy hospital is concerned
15. I would take special care (like wearing gloves and masks) when treating a patient with leprosy
16. It is possible for me to have leprosy if I treat leprosy patients
17. Dressing for a leprosy patient is not disgusting most of the times
18. Working in a leprosy hospital is one of the best ways of exhibiting humanitarian nature
19. If someone in my family has leprosy I would not mind talking about it to my friends
20. I get a sense of satisfaction when I treat patients with leprosy
21. I would be supportive of a person who has leprosy
22. It is possible to manage leprosy like any other disease in the general health service
23. It would be better to treat leprosy patients in general hospitals instead of ‘special’ leprosy hospitals
24. I have no problem in treating leprosy patients with deformities or ulcers
25. I am willing to be involved in diagnosing/managing leprosy cases at my health care facility
26. I don’t mind working in an all leprosy hospital
27. Leprosy is curable

### Validity and reliability

The experts’ group judged the 27 items to measure attitudes towards leprosy at face value thereby establishing face validity. In terms of the content validity, the researchers found that the items in each of the affective, behavioural and cognitive domains were an adequate representation of the construct of attitude. As regards reliability a total of 54 HCPs participated in the reliability exercise (Table 4).

**Table 4:** Number of participants by district in Tamil Nadu.

Category	Number	District
MOs	7	Kancheepuram
HIs	10	Kancheeepuram
HIs	11	Tiruvallur
VHNs	26	Kancheepuram
**Total**	**54**	

While 54 persons participated in the test, thirty-eight persons (70%) completed the re-test. Unfortunately, despite scheduling a date and time for the re-test in consultation with the HCPs, many of them either did not come or else were pre-occupied and could not fill in the questionnaire. The ICC for test re-test reliability of the 27 items scale was 0.6 (95% CI 0.20–0.78) indicating marginal intra-class correlation while the overall Cronbach’s alpha was 0.85 The alphas for each of the affective and behavioural components was good at 0.78 and 0.69 respectively, but the alpha for the cognitive component was low at 0.53.

## Discussion

Understanding attitudes of health care providers towards persons affected by leprosy is an important step towards their better treatment, improved quality of life and mental health. Hence the development of an appropriate measurement of attitude becomes essential. Our use of the ABC model that served as the conceptual framework in guiding the development of the questionnaire was an added strength as evident by the fact that several scales in the field of mental health, disability and prejudice have been developed based on this framework [28–30]. As per the model, only a proper representation of affect, behavior and cognition best explains the construct of “attitude”. Therefore, a good attitude questionnaire needs to have a healthy mix of items that tap into all these three domains. This scale with a total of 27 items (10-Affect, 8-Behaviour, 9-Cognition) has a good balance of these different domains.

While there are other instruments that have been developed to measure the attitudes of HCPs towards leprosy patients, some had been developed in a totally different cultural context, some were lacking in a comprehensive assessment of the “attitude construct” and others were assessing knowledge rather than attitude. For example, Rao et al assessed knowledge, attitude and practice of MOs towards leprosy using a questionnaire that they had developed [22]. Their 58 items questionnaire had only 6 questions on attitudes with inadequate inclusion of affective, behavioural and cognitive components. Furthermore, the response scale, they used included only ‘yes’ or ‘no’ options. Use of Likert type of response scales on the other hand, would have been more appropriate and would have provided a diverse range of options in keeping with the measurement of attitudes. Attitude response scales are ideally required to indicate the direction (positive or negative) and the intensity (very likely to somewhat likely, for example) of those attitudes [31].

Cultural validation plays an important role in providing accurate estimates of the true attitudes held by individuals in a certain cultural context [32]. This in turn would provide cues as to the nature of remedial measures that would be needed to inculcate a more positive outlook among HCPs towards persons affected by leprosy. Content validity of the scale was enhanced through a process of triangulation, which included reviews of existing scales that measured attitudes of HCPs towards various disabilities and qualitative interviews with different categories of HCPs. The qualitative interviews helped in understanding provider perceptions with regard to treating leprosy patients in the Indian care setting and informed the framing of items that were included in the scale.

Thurstone scaling technique which is based on the consensus scale approach was used for selecting items [26]. Having a panel of experts evaluate each item in terms of whether it is relevant to the topic area and unambiguous in implication helped to enhance the comprehensiveness of the scale. It has been argued that the values assigned to various statements by experts may in effect reflect their own attitudes, and therefore is not entirely objective. We attempted to overcome this by including experts from diverse fields, namely, community medicine, psychology, psychiatry and social science who brought in diverse perceptions based on their respective experiences.

Stigma results in delayed diagnosis and might have an effect on health seeking behavior [33]. Inequalities due to the social, cultural, and economic context in these vulnerable segments act as barriers to access health services [34]. Hence, it is important to be able to assess this type of stigma for the prevention and management of NTDs like leprosy. Assessing stigma is not an easy task. There are several instruments available, but these were developed with different aims or tested in different settings [35–37]. Where negative attitudes prevail, the importance of carrying out interventions that will educate and sensitize HCPs against the harms that such attitudes can cause could be one strategy that could be implemented [38]. Hovland et al. have shown that when there is recurrent persuasive communication, it could result in change of attitudes and opinions. Further the study has shown that the characteristics of the person who receives the communication, the person who delivers the communication, the characteristic of the messages, source of the messages etc. are factors that influence attitude change [39]. Integration of leprosy programmes into general health care, Information Education & Communication programmes and socio-economic rehabilitation strategies have been found to have some effect in stigma reduction in leprosy [40]. But what needs to be kept in mind is that educating civil society needs to be a continuous process. Simply running a few education sessions tends to have a low success rate. Interventions like social marketing have been proved by Brown et al, to be an effective co-adjunct to reducing leprosy related stigma [41]. In Indonesia, contact intervention was found effective in increasing knowledge about leprosy and the negative attitudes reduced. [42]. These interventions target the health system, and community, including persons affected by leprosy and thus provide evidence of strategies that could be tested in the Indian context.

In terms of limitations, the test re-test reliability of the instrument was only marginal. The test was carried out during the monthly review meetings when all the different cadres of HCPs usually gathered at the block PHC. A total of 54 HCPs thus completed the test. The re-test had to be done between 12–14 days of the test and many HCPs who had participated in the test were otherwise preoccupied and indicated that they lacked the time to complete the scale again. The inadequate sample size could have contributed to the marginal test re-test reliability estimates. The overall Cronbach’s alpha for the scale was very good at 0.85 with both the affective and behavioural components showing good internal consistency. However, the alpha for the cognitive component was poor at 0.53. Tavakol and Dennick (2011) have described that one of the reasons for poor alphas in a scale can be attributable to poor inter relatedness between items. Future research will need to re-examine the items under the cognitive component and make suitable modifications such that it is more homogenous [43].

In terms of future work, administering the scale to a larger number of HCPs working in primary care settings will be helpful. One other limitation was our inability to conduct a factor analysis because of the small sample size. A larger sample would have allowed us to discover the underlying factor structure of the questionnaire and determine whether it indeed conforms to the domains we developed based on the ABC model. Factor analysis would also be an effective method for further scale reduction, thereby making it more clinic friendly.

### Conclusions

Despite some limitations, this scale developed to measure the attitudes of HCPs towards people affected by leprosy presents a first step in this direction. It could be used to evaluate provider attitudes and could aid in identifying positive or negative attitudes held by providers towards persons affected by leprosy because negative attitudes may impede leprosy control activities. It may also serve as a viable tool to assess changes in the attitudes of HCPs following an intervention. However, further research is required, in terms of using the scale on a much larger sample of HCPs across different parts of India so as to fully substantiate its relevance and cultural appropriateness. With some adaptations, the scales can also be validated for other NTDs which are endemic in India.

## Supporting information

S1 File COREQ checklist. (DOCX)

S2 File Interview and FGD guide. (DOCX)

S3 File Quantitative data. (XLSX)
