# Supplementary material for: Development and validation of a scale to assess attitudes of health care providers towards persons affected by leprosy in southern India
Source: PLoS Negl Trop Dis. 2018 Sep 25;12(9):e0006808. doi: 10.1371/journal.pntd.0006808 (PMC6177202; doi:10.1371/journal.pntd.0006808)
Supplement: S1 File — (DOCX) [file pntd.0006808.s001.docx]

**Consolidated criteria for reporting qualitative studies (COREQ): 32-item checklist**

Developed from:

Tong A, Sainsbury P, Craig J. Consolidated criteria for reporting qualitative research (COREQ): a 32-item checklist for interviews and focus groups. *International Journal for Quality in Health Care*. 2007. Volume 19, Number 6: pp. 349 – 357

**YOU MUST PROVIDE A RESPONSE FOR ALL ITEMS. ENTER N/A IF NOT APPLICABLE**

| **No. Item** | **Guide questions/description** | **Reported on Page # and Line#** |
| --- | --- | --- |
| **Domain 1: Research team and reﬂexivity** |  |  |
| *Personal Characteristics* |  |  |
| 1. Inter viewer/facilitator | Which author/s conducted the inter view or focus group? | **Results:** Shuba Kumar (SK) & Rani Mohanraj (RM) did all the interviews with the healthcare providers and their senior research assistant (SRA) carried out the focus groups |
| 2. Credentials | What were the researcher’s credentials? E.g. PhD, MD | **Method**s :Both SK and RM hold a Ph.D while their SRA has an M.Sc degree |
| 3. Occupation | What was their occupation at the time of the study? | **Methods: SK is a social scientist, RM is a psychologist** and the SRA is a Project Coordinator |
| 4. Gender | Was the researcher male or female? | All researchers are female |
| 5. Experience and training | What experience or training did the researcher have? | **Methods**: SK has over 20 years of research experience Has been trained in epidemiology and social science under the International Clinical Epidemiology Network in the University of Newcastle, Australia  RM underwent a three month training as a Fogarty Fellow in the University of Washington, Seattle, USA and has over 15 years of research experience  SRA has been trained by SK and RM and has over 10 years of research experience |
| *Relationship with participants* |  |  |
| 6. Relationship established | Was a relationship established prior to study commencement? | No relationship established |
| 7. Participant knowledge of the interviewer | What did the participants know about the researcher? e.g. personal goals, reasons for doing the research | Participants did not know the researcher |
| 8. Interviewer characteristics | What characteristics were reported about the inter viewer /facilitator? e.g. Bias, assumptions, reasons and interests in the research topic | **Method**s: None of the characteristics of the interviewer/facilitator were reported, and the only thing informed was that GLRA is conducting the study to assess attitude of HCP towards leprosy/ |
| **Domain 2: study design** |  |  |
| *Theoretical framework* |  |  |
| 9. Methodological orientation and Theory | What methodological orientation was stated to underpin the study? e.g. grounded theory, discourse analysis, ethnography, phenomenology, content analysis | **Methods:** We used a framework analytical approach in analyzing the qualitative data. The main aim was to derive the items that would inform the development of the scale. Using qualitative methods of interviews and FGDs with the target population was one method of ensuring good content validity of the scale. We used the ABC model (Affect, Behaviour, and Cognition) that best explains the ‘attitude’ construct to guide us in scale development |
| *Participant selection* |  |  |
| 10. Sampling | How were participants selected? e.g. purposive, convenience, consecutive, snowball | **Method**s: Participants were selected purposively |
| 11. Method of approach | How were participants approached? e.g. face-to-face, telephone, mail, email | **Methods**: Participants were first contacted over phone and after obtaining their oral consent, we met with them at their health centres and obtained their written consent before including them in the study |
| 12. Sample size | How many participants were in the study? | **Results:** A total of 10 semi structured interviews 5 each were carried out with Health Inspectors and medical officers. Two focus group discussion were carried out with Village Health Nurses (10 in a group) |
| 13. Non-participation | How many people refused to participate or dropped out? Reasons? | **Methods**: There were no drop-outs or refusals for the qualitative data collection |
| *Setting* |  |  |
| 14. Setting of data collection | Where was the data collected? e.g. home, clinic, workplace | **Methods:** All data collection was done in the Primary Health Centres to which the medical officers, health inspectors and village nurses were attached. |
| 15. Presence of non-participants | Was anyone else present besides the participants and researchers? | **Results:** All interviews and FGDs were carried out in privacy with none other than the research team member present during the session. |
| 16. Description of sample | What are the important characteristics of the sample? e.g. demographic data, date | **Results:** Five MOs and 5 HIs participated in the qualitative interviews. While three MOs and three HIs were from Villupuram, two MOs and two HIs were from Kancheepuram. One MO and three HIs were women, all the rest were men. The MOs had all completed their MBBS degree. While four of the HIs held post graduate degrees one had only completed schooling (12 years). Two FGDs were carried out with the VHNs, one in Kancheepuram and one in Villupuram. The VHNs were all women who had completed their schooling. |
| *Data collection* |  |  |
| 17. Interview guide | Were questions, prompts, guides provided by the authors? Was it pilot tested? | **Methods:** We developed interview and focus group guides but probes and additional questions over and above what was stated in the guide were used. No pilot testing was done for the qualitative component of study |
| 18. Repeat interviews | Were repeat inter views carried out? If yes, how many? | N/A |
| 19. Audio/visual recording | Did the research use audio or visual recording to collect the data? | **Method:** All interviews and FGDs were audio recorded |
| 20. Field notes | Were ﬁeld notes made during and/or after the inter view or focus group? | **Methods**: Field notes were made both during and after the interviews and FGDs and included summary of important issues that emerged during the interviews/FGDs and other thoughts relevant to the study |
| 21. Duration | What was the duration of the inter views or focus group? | **Method**s: Each interview was approximately for about 45 minutes while the FGDs were for an hour or more |
| 22. Data saturation | Was data saturation discussed? | **Methods**: Yes |
| 23. Transcripts returned | Were transcripts returned to participants for comment and/or correction? | Not returned |
| **Domain 3: analysis and ﬁndings** |  |  |
| *Data analysis* |  |  |
| 24. Number of data coders | How many data coders coded the data? | **Methods**: Only SK and RM coded the data |
| 25. Description of the coding tree | Did authors provide a description of the coding tree? | N/A |
| 26. Derivation of themes | Were themes identiﬁed in advance or derived from the data? | **Method**s: Themes were identified in advance namely, Affect, Behaviour and Cognition (ABC model) and we specifically looked for these issues in the interviews/FGDs |
| 27. Software | What software, if applicable, was used to manage the data? | **NVivo:** No software was used. |
| 28. Participant checking | Did participants provide feedback on the ﬁndings? | **Strengths and limitations**: Yes participants did provide feedback on the draft scale that was developed |
| *Reporting* |  |  |
| 29. Quotations presented | Were participant quotations presented to illustrate the themes/ﬁndings? Was each quotation identiﬁed? e.g. participant number | **Results**: Yes quotations have been presented and identified by category, place and participant number |
| 30. Data and ﬁndings consistent | Was there consistency between the data presented and the ﬁndings? | **Relationship to existing knowledge**: Yes |
| 31. Clarity of major themes | Were major themes clearly presented in the ﬁndings? | **Results**: The use of qualitative methods was resorted to, primarily to inform the development of the scale. Therefore, the qualitative findings are not presented as themes. Our aim was to identify perceptions of HCPs that best reflected the affect, behaviour and cognition component of the attitude construct |
| 32. Clarity of minor themes | Is there a description of diverse cases or discussion of minor themes? | **Discussion**: NA |

**Once you have completed this checklist, please save a copy and upload it as part of your submission. When requested to do so as part of the upload process, please select the file type: *Checklist*. You will NOT be able to proceed with submission unless the checklist has been uploaded. Please DO NOT** **include this checklist as part of the main manuscript document. It must be uploaded as a separate file.**
