# Supplementary material for: Development and validation of a scale to assess attitudes of health care providers towards persons affected by leprosy in southern India
Source: PLoS Negl Trop Dis. 2018 Sep 25;12(9):e0006808. doi: 10.1371/journal.pntd.0006808 (PMC6177202; doi:10.1371/journal.pntd.0006808)
Supplement: S2 File — (DOCX) [file pntd.0006808.s002.docx]

**Interview Guide with HCPs-Doctors, HIs**

**FGDs with VHNs**

**Demographic Information**

1. Name
2. Age 2.1 Gender
3. Education
4. Current position in health facility
5. Number of years of overall work experience
6. Number of years in current facility
7. Can you describe your roles and responsibilities with regard to care and management of leprosy patients?
8. How big a problem is leprosy in your district/ health facility? On what basis do you state this. (*Probe: how many cases does he/she see in a week/month*)
9. Usually at what stage of the disease do the patients usually come to your clinic? Do they report with disability? (*Probe: is it usually at an advanced stage of the disease, if so why don’t they comeearlier, is it because of poor awareness, issues concerning stigma etc)*
10. What according to you are the common modes of disease transmission?
11. How do you feel about working with leprosy patients? *(Probe: their attitudes towards persons with leprosy)*

What is your belief about chronic diseases such as HIV/TB/Leprosy patients in general? Anything about leprosy patients in particular? Do those having ulcers or other complications report to you for management?

1. What do you see as some challenges in working with leprosy patients and how do you deal with this? *(Probe: concerns regarding being at risk or becoming infected, issues concerning stigma)*
2. How much do you think issues concerning stigma come in the way of patients seeking care in general health care settings, please describe. *(Probe: patient fears of being stigmatized/discriminated against in the health facility, in the community etc)*
3. Do you believe that patients with leprosy face discrimination in health care settings, if so what form does this discrimination take and why do you thinks this happens? *(Probe: fears of contagion, whether they feel disgust at having to treat such patients etc*)
4. Do you think health care providers are justified in feeling this way towards leprosy patients, why?
5. What suggestions do you have towards reducing this stigma of health care providers (towards patients) with leprosy?
